# Oncologic outcomes after Total Mesometrial Resection (TMMR) or treatment according to current international guidelines in FIGO (2009) stages IB1-IIB cervical cancer: an observational cohort study

**DOI:** 10.1016/j.eclinm.2024.102696

**Published:** 2024-06-20

**Authors:** Henrik Falconer, Anna Norberg-Hardie, Sahar Salehi, Emilia Alfonzo, Laura Weydandt, Nadja Dornhöfer, Benjamin Wolf, Michael Höckel, Bahriye Aktas

**Affiliations:** a Department of Pelvic Cancer, Karolinska University Hospital and the Department of Women's and Children's Health, Karolinska Institutet, Stockholm, Sweden; b Centre for Clinical Research Sörmland, Uppsala University, Eskilstuna, Sweden; c Department of Obstetrics and Gynaecology, Sahlgrenska University Hospital and Department of Obstetrics and Gynaecology, Institute of Clinical Sciences, Sahlgrenska Academy, University of Gothenburg, Gothenburg, Sweden; d Department of Gynecology, University Hospital Leipzig and Leipzig School of Radical Pelvic Surgery, University of Leipzig, Leipzig, Germany

**Keywords:** Cervical cancer, Cancer field surgery, Radical hysterectomy, TMMR

## Abstract

**Background:**

According to international guidelines, standard treatment (ST) with curative intent in cervical cancer (CC) comprises radical hysterectomy and pelvic lymphadenectomy in early stages (International Federation of Gynecology and Obstetrics (FIGO) 2009 IB1, IIA1), adjuvant chemoradiation is recommended based on risk factors upon final pathology. Definitive chemoradiation is recommended in locally advanced stages (FIGO 2009 IB2, IIA2, IIB). Total mesometrial resection (TMMR) with therapeutic lymph node dissection (tLND) without adjuvant radiation has emerged as a promising treatment. Here we compare oncologic outcome by TMMR + tLND or ST.

**Methods:**

In this observational cohort study, women treated according to international guidelines were identified in the population-based registries from Sweden and women treated with TMMR were identified in the Leipzig Mesometrial Resection (MMR) Study Database (DRKS 0001517) 2011–2020. Relevant clinical and tumour related variables were extracted. Recurrence-free survival (RFS) and overall survival (OS) by ST or TMMR was analysed with log-rank test, cumulative incidence function and proportional hazard regression yielding hazard ratios (HR) with 95% confidence intervals (CI), adjusted for relevant confounders.

**Findings:**

Between 2011 and 2020, 1007 women were included in the final analysis. 733 women were treated according to ST and 274 with TMMR. RFS at five years was 77.9% (95% CI 74.3–81.1) and 82.6% (95% CI 77.2–86.9) for the ST and TMMR cohorts respectively (p = 0.053). In early-stage CC, RFS was higher after TMMR as compared to ST, 91.2% *vs* 81.8% (p = 0.002). In the adjusted analysis, TMMR was associated with a lower hazard of recurrence (HR 0.39; 95% CI 0.22–0.69) and death (HR 0.42; 95% CI 0.21–0.86) compared to ST. The absolute difference in risk of recurrence at 5 years was 9.4% (95% CI 3.2–15.7) in favor of TMMR. In locally advanced CC, no significant differences in RFS or OS was observed.

**Interpretation:**

Compared to ST, TMMR without radiation therapy was associated with superior oncologic outcomes in women with early-stage cervical cancer whereas no difference was observed in locally advanced disease. Our findings together with previous evidence suggest that TMMR may be considered the primary option for both early-stage and locally advanced cervical cancer confined to the Müllerian compartment.

**Funding:**

This study was supported by grants from Centre for Clinical Research Sörmland (Sweden) and Region Stockholm (Sweden).

**Research in context**

**Evidence before this study**

Results from the Leipzig Mesometrial Resection Study, a prospective single-center study, have repeatedly indicated similar or superior oncologic outcomes after cancer field surgery for cervical cancer without the need for radiation therapy. We performed a systematic review (according to the PRISMA guidelines) on oncologic outcomes after total mesometrial resection (TMMR) and therapeutic lymphadenectomy (tLND) in comparison with treatment according to international guidelines (standard treatment) for cervical cancer FIGO (2009) stages IB1-IIB. We searched MEDLINE, Embase, and Scopus using terms for uterine cervical neoplasms combined with terms for TMMR, compartment-based surgery, embryologically based resection, and cancer field surgery (no restrictions were applied for publication date, study design or language). No studies were identified in the systematic review.

**Added value of this study**

To our knowledge, this large observational cohort study is the first study on this topic to date, including more than 1000 patients treated either with TMMR or according to standard treatment. We show that TMMR + tLND was associated with superior oncologic outcomes compared to standard treatment for FIGO (2009) stages IB1 and IIA1 cervical cancer whereas no difference was observed for recurrence-free or overall survival in women with locally advanced disease (stages IB2, IIA2 and IIB). No women received primary or adjuvant radiation therapy in the TMMR cohort.

**Implications of all the available evidence**

The current results in combination with previous findings suggest that TMMR could be considered as standard treatment for early-stage cervical cancer and for locally advanced cervical cancer confined to the Mullerian compartment. Further, a shift in treatment strategy could avoid radiation therapy, which may confer improved postoperative morbidity and quality of life and radiation therapy could be spared for salvage treatment.

## Introduction

Treatment strategies for curable cervical cancer are mainly based on tradition rather than scientific evidence. The concept of “wide surgical excision” as opposed to organ restricted resection was first described in the early 1900s by Ernst Wertheim, demonstrating superior oncologic outcomes when tissue adjacent to the uterine cervix and upper vagina was removed together with the uterus.^1^ The so-called radical hysterectomy or Wertheim's operation has since been the mainstay of surgical treatment of cervical cancer confined to the pelvis. With the introduction of efficient radiation therapy in the 1920s, an additional treatment option became available. In early-stage cervical cancer, treatment schemes combining these modalities have since been commonly applied if certain tumour specific risk factors are present at final pathology after surgery, suggesting that unimodal treatment is insufficient for adequate pelvic control.

Current international guidelines are primarily based on retrospective case series and a small number of outdated randomised controlled trials. The stage-dependent treatment recommendations, with surgery advised for early-stage and radiation therapy for locally advanced disease, may be considered too simplistic, suggesting that early stages of cervical cancer cannot be controlled with surgical resection alone or that locally advanced cervical cancer is inoperable.^2^ The classical radical hysterectomy is poorly defined, resulting in several different classification systems and subsequent interpretations thereof.^3^, ^4^, ^5^, ^6^

The inconsistencies in primary treatment options suggests that the underlying locoregional pattern of dissemination in cervical cancer is underexplored. Historically, textbook anatomy and surgical dissection artefacts have been the determinants of treatment. In rectal cancer, the introduction of compartmental principles for primary surgery represented a paradigm shift in the 1990s with a dramatic improvement in local control.^7^ Anatomical compartments also constitute the basis of the cancer field model proposed by Höckel et al., challenging the prevailing random spread concept.^8^^,^^9^ The ontogenetic cancer field model postulates that (1) cancer spreads within anatomical compartments determined by the ontogenesis of the normal tissue from which it originated, (2) malignant progression of the cancer cells is inversely related to the fate progression of the normal cell type with regard to the cells’ colonization potential. As a consequence, local cancer staging and treatment should be based on ontogenetic instead of traditional anatomy. For the regional spread of carcinomas, the cancer field model claims pathological mechanisms of peripheral immune tolerance. Considering locoregional morphologic links of the lymphatic system derived from its ontogenesis, first-, second- and third-line lymph node surveillance regions are topographically defined for an individual local tumour. The clinical translation of the cancer field model introduces ontogenetic staging and cancer field resections with therapeutic lymph node dissection as novel surgical techniques to treat carcinomas without adjuvant radiation therapy.

The morphological insights gained from the studies of the ontogenesis of the Mullerian system in human females led to the development of surgical techniques designated as mesometrial resections. Total and extended mesometrial resection (TMMR, EMMR) with therapeutic lymph node dissection (tLND) refer to the cancer field surgery for cervical and vaginal carcinomas.^8^^,^^10^ The cancer field model has been clinically substantiated by single-centre prospective data from more than 600 consecutive surgical procedures performed at the University Hospital of Leipzig, Germany demonstrating excellent local control for both early and locally advanced cervical cancer, despite completely omitting radiation therapy.^11^ These results were recently reproduced in a multi-centre European prospective observational study.^12^ However, the oncologic outcomes after TMMR have not been subjected to comparison to women treated according to universally accepted guideline recommendations. The complexity behind the cancer field model may have deterred surgeons from adopting the technique and the uptake of TMMR has been limited. Accordingly, a fundamental prerequisite for testing in a phase III trial is lacking. For this reason, we investigate oncologic outcomes after TMMR as compared to treatment based on current guideline recommendations in this observational cohort study.

## Methods

### Study design and patients

This was an observational cohort study comparing oncologic outcomes after TMMR or standard treatment (ST) with curative intent in women with cervical cancer FIGO (2009) stages IB1-IIB. All data were collected prospectively whereas the analyses were conducted in retrospect. Exposed women were subjected to surgical treatment with TMMR within the Leipzig Mesometrial Resection Study, a prospective single-center study, between January 2011 and December 2020. Unexposed comprised all women from two health care regions in Sweden subjected to standard international guideline-based treatment during the same period. The time period was chosen based on the start of the string “cervix” in the Swedish Quality of Gynecologic Cancer (SQRGC) and to ensure an adequate sample size.

Swedish data from the Region of Stockholm/Gotland and the Western Health Care Region represented the standard guideline-based treatment and was retrieved from the population-based Swedish Quality Registry for Gynecologic Cancer (SQRGC). The SQRGC started in 2008 and string cervical cancer was added to the registry in 2011. Data entry was prospective and includes patient, tumour- and treatment characteristics as well as follow-up data and outcomes including vital status. The SQRGC has been independently validated and has a coverage of approximately 95% relative to the National Cancer Register (NCR).^13^ Reporting to the NCR is compulsory and mandated by Swedish law. Furthermore, SQRGC is linked to the Swedish Population Registry, enabling daily updates on vital status. Data on patients treated with TMMR were obtained from the Leipzig Mesometrial Resection Study, a prospective single-center study. The details of this study including description of the TMMR procedure have been described elsewhere.^10^^,^^14^

The following variables were extracted from the two databases: Age at diagnosis, stage according to FIGO 2009, stage according to the Tumour Node Metastasis (TNM) classification, tumour size including the categories; <20 mm, 20–40 mm, and >40 mm, histologic subtype, lymph vascular space invasion (LVSI), primary treatment, type of surgical treatment (radical hysterectomy by minimally invasive, radical hysterectomy by laparotomy or TMMR), type of oncological primary treatment, adjuvant treatment, type of adjuvant treatment, external beam radiation therapy (EBRT), number of chemotherapy cycles, lymph node status and relapse type. In the ST cohort, no surgico-pathological staging was available in women with locally advanced cervical cancer, tumour size and pathological lymph nodes were assessed by imaging or by histopathology if a biopsy from lymph nodes had been performed.

Patients were eligible for analysis if they were 18 or older, had a diagnosis of cervical cancer (squamous, adeno- or adeno-squamous carcinomas) between 2011 and 2020 with presumed FIGO (2009) stage IB1-IIB before treatment. Patients were excluded if they had other primary malignancies than cervical cancer, other histologic subtypes than squamous, adeno- or adeno-squamous carcinomas, treated with neoadjuvant chemotherapy, were pregnant at diagnosis and/or treatment, treated with palliative intent, declined treatment, had synchronous carcinomas or did not receive treatment according to standard.

Standard guideline-based treatment comprised radical hysterectomy and pelvic lymphadenectomy in FIGO (2009) stages IB1 and IIA1, followed by adjuvant chemoradiation according to the histopathological risk criteria if indicated.^2^^,^^15^ In locally advanced disease (FIGO2009 stages IB2, IIA2 and IIB), women received external beam radiation therapy (EBRT) to the pelvis with concomitant chemotherapy followed by two-dimensional (2D) or three-dimensional (3D) MRI-guided brachytherapy. Extended field paraaortic radiation therapy was applied in case of nodal involvement on imaging. EBRT techniques comprised intensity-modulated radiation therapy (IMRT) and volumetric arc therapy (VMAT) with CT-based treatment planning. The EBRT dose was 45–50 Gy in 1**.**8–2 Gy fractions. Concomitant chemotherapy was weekly intravenous cisplatin 40 mg/m^2^, 5–6 cycles, 1 day per cycle.

Total mesometrial resection (TMMR) comprises surgical excision of the local tumour with regard to its ontogenetic stage-associated cancer field and therapeutic lymphadenectomy (tLND) of both basin and intercalated nodes.

### Outcomes

The primary outcome was recurrence-free survival (RFS) in all, early-stage and locally advanced cervical cancer. RFS was defined as the time from the first day of treatment to the date of recurrence (both local and distant) or death or until the 25th of August 2022 in the ST group (unexposed cohort) and until the 20th of December 2022 in the TMMR group (exposed cohort). Secondary outcome was overall survival (OS), defined as the time from the day of first treatment to the date of death (or until the 25th of August 2022 in the ST group (unexposed cohort) and until the 20th of December 2022 in the TMMR group (exposed cohort). Confounding variables were predefined and chosen based on known clinical association with the outcomes: age, year of treatment, stage, histology, lymph node metastasis and tumour size.

### Ethics

The study reporting adheres to the Strengthening the Reporting of Observational Studies in Epidemiology (STROBE) guidelines and was approved by the ethical review board Dnr-2021-03261 in Sweden and the MMR-study was approved by the Ethics Committee of the University of Leipzig (012/13-28012013; 171-2006; 192/2001; 151/2000) and registered with the German Cancer Registry (DRKS 0001517). Informed consent was obtained from all participants in Leipzig Mesometrial Resection Study whereas the need for informed consent was waived by the ethical review board in Sweden as only pseudoanonymized data were used in the analyses.

### Statistics

Descriptive statistics is presented with numbers and proportions, medians and interquartile ranges (IQR) or means and standard deviations (SD) as appropriate. Distributional differences were tested using Fisher's exact test for categorical variables and Mann–Whitney test for continuous variables. Time for recurrence-free survival (RFS) was calculated from the date of treatment to the date of local recurrence, date of distant recurrence or date of death, whichever came first. For event-free patients, time was calculated from the date of treatment to the date of last follow-up. Time for overall survival (OS) was calculated from the date of treatment to date of death, or for patients still alive, to the date of last follow-up. The median follow-up time was estimated using the reversed Kaplan–Meier method. RFS and OS were estimated and graphically displayed using the Kaplan–Meier method. Time to first event (local, distant or death) were estimated and plotted using nonparametric cumulative incidence functions, taking the competing risks into account. Differences between the study groups in survival and cumulative incidence at 5 years were supplemented with Wald-type 95% confidence intervals. The modelling of hazards–when competing risks were absent (RFS, OS)–was performed using proportional hazards regression with results presented as hazard ratios (HR) together with 95% confidence intervals and Wald p-values. In the models where competing risks were present—first event (local, distant or death)—subdistribution hazards were modelled using competing risk regression. Results from these models are presented as subdistribution hazard ratios (sHR) together with 95% confidence intervals and Wald p-values. Models were adjusted for known risk factors for recurrence and/or death and multivariate analysis of confounders is presented in Supplementary data (Table 2). The assumption of proportional hazards/subdistribution hazards were evaluated by including covariate∗time interactions into the regression models. To assess potential residual confounding in the total population, propensity score matching (PSM) and inversed probability weighting (IPW) analyses were performed. The potential impact of immortal time bias (ITB) in women treated with primary radiation therapy was analysed using landmark analysis. To account for the longer duration of radiation therapy, 180 days were added to the standard treatment in the landmark analysis. The statistical analyses were done using Stata (StataCorp. 2023. Stata Statistical Software: Release 18. College Station, TX: StataCorp LLC).

### Role of funding source

Funding sources had no role in the collection, analysis and interpretation of data.

## Results

**Fig. 1:**
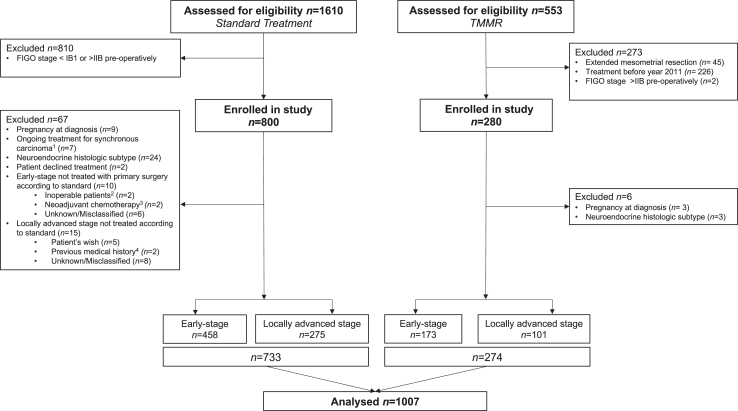
**Flow chart**. Abbreviations: TMMR, Total MesoMetrial Resection.^1^ Ovarian cancer *(n* = 3). Breast cancer *(n* = 2), Colon cancer *(n* = 1) Thyroid cancer *(n* = 1). ^2^Women seen as inoperable due to; age (*n* = 1), comorbidity, ASA 3 (*n* = 1), Unknown (*n* = 3) ^3^Neoadjuvant treatment give due to; primary histopathology showing glassy cell tumour but final pathology showing squamous (*n* = 2) ^4^Earlier severe psychosis, not eligible for radiation therapy (*n* = 1), previous chemoradiation therapy *(n* = 1).

Between 2011 and 2020, 2163 women were assessed for eligibility and 1080 women were enrolled in the study (280 women treated with TMMR, and 800 women treated according to ST), see study flow chat in Fig. 1). In the TMMR cohort, six women were excluded; pregnancy at diagnosis and treatment (*n* = 3), histologic subtype (*n* = 3). In the ST group 67 women were excluded; pregnancy at diagnosis (*n* = 9), synchronous carcinoma (*n* = 7), histologic subtype (*n* = 24), patient declined treatment (*n* = 2) and treatment not according to standard (*n* = 25) (Fig. 1). In total 1007 women were included in the final analyses (TMMR *n* = 274, ST *n* = 733).

The distribution of patient-, tumour- and treatment characteristics are presented in Table 1. There were no distributional differences in age, stage, histologic subtype, tumor size or presence of lymph node metastasis between the groups (Table 1). In the TMMR group, a higher proportion of women had lymph node metastasis diagnosed based on gold standard surgical resection with histopathology (100% *vs* 25%) and data on the presence of lymph vascular space invasion. Moreover, no women in the TMMR cohort received primary chemoradiation therapy (0 *vs* 38%) or adjuvant radiation therapy (0 *vs* 18%), see Table 1.

**Table 1:** Patient-, tumour and treatment characteristics of women with cervical cancer treated with curative intent by total mesometrial resection or according to standard treatment 2011 to 2020.

	TMMR *n* = 274	Standard Treatment *n* = 733	p-value^a^
**Age, years**			
Median			
IQR	44 (36–55)	45 (38–55)	0.237^b^
**FIGO (2009) stage, no. (%)**			
IB1	164 (60)	449 (61)	0.001
IB2	25 (9)	70 (10)	
IIA1	9 (3)	9 (1)	
IIA2	2 (1)	40 (5)	
IIB	74 (27)	165 (23)	
**Early stage, no. (%)**			
IB1, IIA1	173 (63)	458 (62)	0.553
**Locally advanced stage, no. (%)**			
IB2, IIA2, IIB	101 (37)	275 (38)	0.553
**Histologic subtype, no. (%)**			
Squamous Cell Carcinoma	201 (73)	483 (66)	0.063
Adenocarcinoma	64 (23)	226 (31)	
Adeno-squamous carcinoma	9 (3)	22 (3)	
Missing		2 (0)	
**Tumor size, millimetres, no. (%)**			
<20	82 (30)	272 (37)	0.082
20–40	110 (41)	244 (33)	
>40	79 (29)	206 (28)	
Missing	3 (1)	11 (2)	
**Lymph nodes metastasis, no. (%)**	80 (29)	218 (30)	0.938
**Lymph node metastases defined by**			
Histopathology	80 (100)	54 (25)	<0.001
Imaging	0 (0)	164 (75)	
**LVSI, no. (%)**	172 (63)	177 (24)	<0.001
Missing	1 (1)	350 (48)	
**Primary treatment, no. (%)**			
Chemoradiation	0 (0)	275 (38)	<0.001
Surgery	274 (100)	328 (45)	
Surgery and adjuvant (chemo)radiation	0 (0)	130 (18)	
**Type of surgery, no. (%)**			
Radical hysterectomy with pelvic LND	0 (0)	458 (100)	<0.001
Total Mesometrial Resection	274 (100)	0 (0)	

**Abbreviations:** TMMR, Total mesometrial resection; IQR, Inter Quartile Range; FIGO, International Federation of Gynecology and Obstetrics; LVSI, Lymph Vascular Space Invasion; LND, Lymphadenectomy.

a Fisher's exact test if not stated otherwise.

b Mann Whitney U test.

### Recurrence-free survival

At a median follow-up time of 5.2 years (IQR 3.1–7.6 years), 22/274 (8%) patients in the TMMR cohort developed pelvic locoregional recurrence. 11/274 (4%) had isolated distant recurrences and 7/274 (3%) had combined pelvic and distant recurrences. In the ST cohort, 80/733 (11%) had a pelvic locoregional recurrence and 50/733 (7%) were diagnosed with a distant or combined recurrence.

**Fig. 2:**
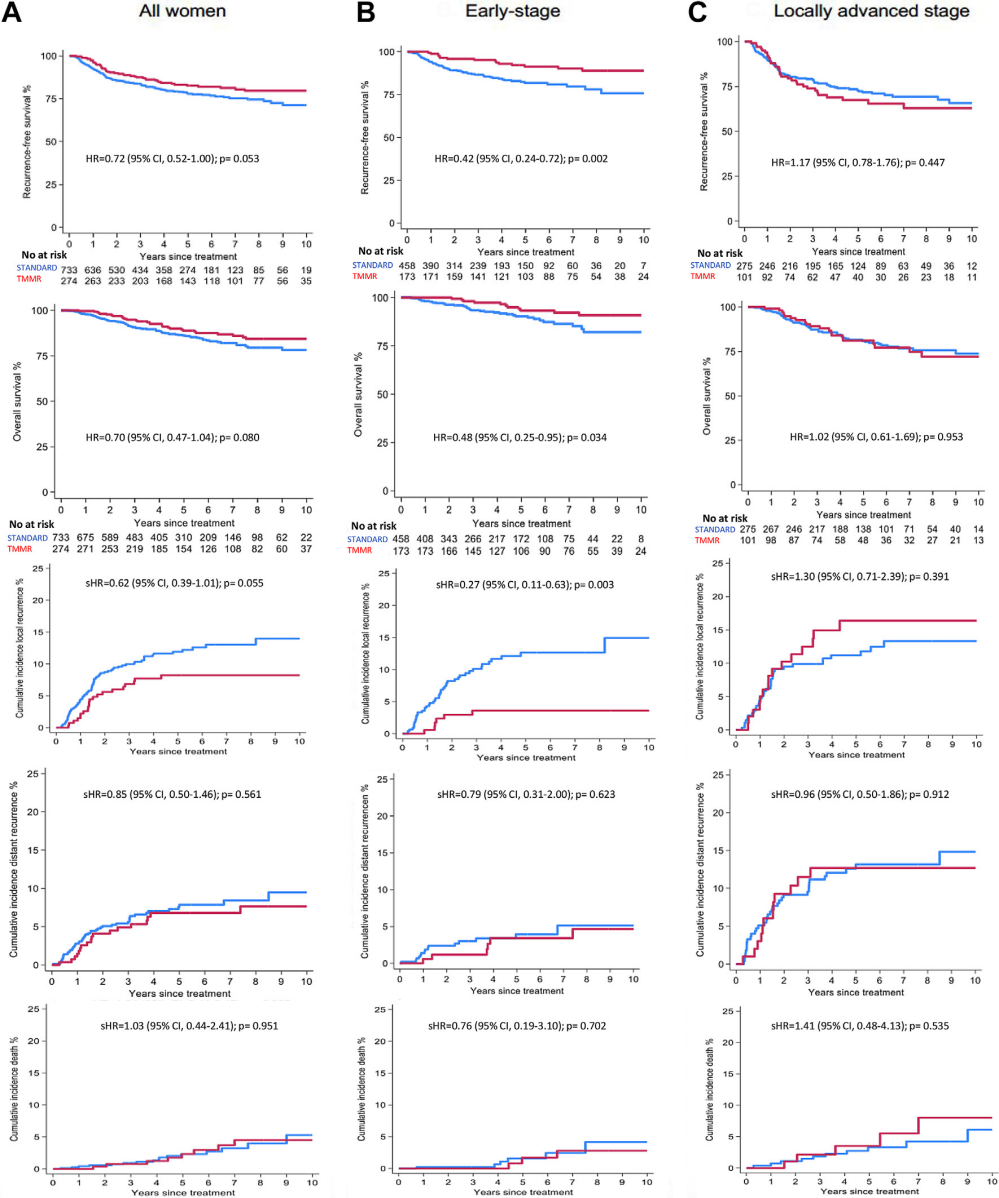
**Recurrence-free and overall survival at 5 years**. Blue line: ST. Red line: TMMR. A: All women. B: Women with early-stage (FIGO∗ IB1 and IIA1). C: Women with locally advanced stage (FIGO∗ IB2, IIA2 and, IIB). Abbreviations TMMR; Total MesoMetrial Resection, ST; Standard Treatment, HR; Hazard Ratio, sHR; subHazard Ratio CI; Confidence Interval, FIGO; International Federation of Gynecology and Obstetrics. ∗Staging according to International Federation of Obstetrics and Gynecology, FIGO, 2009 staging manual.

**Fig. 3:**
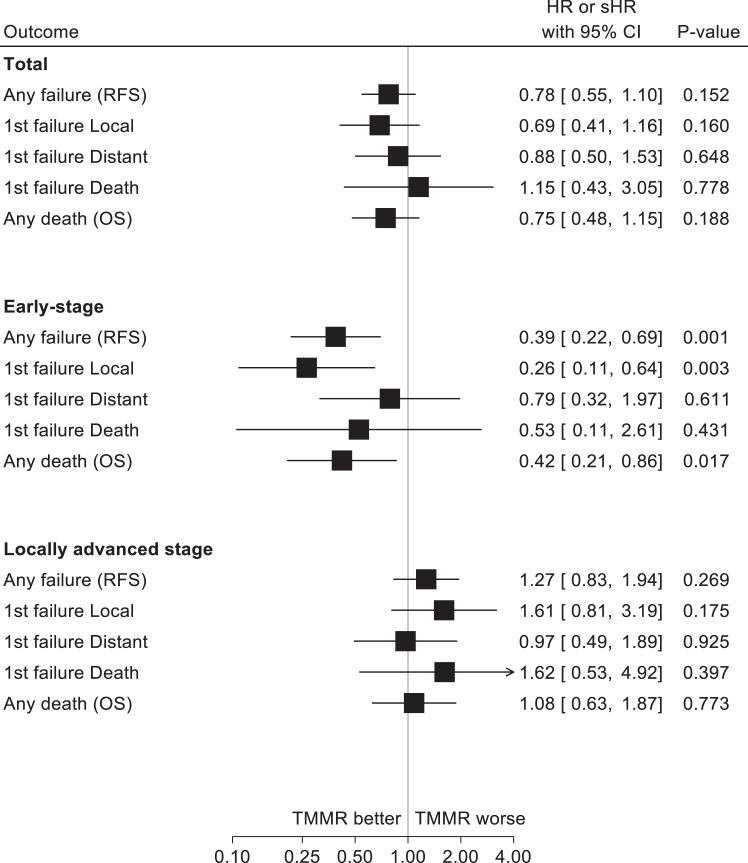
**Forest Plot**. Forest plot of multivariable Cox regression of risk of event by TMMR or Standard Treatment. Adjusted for; age, year of treatment, stage, histology, lymph node metastasis and tumour size. Total: all women. Early-stage: Women with early-stage (FIGO∗ IB1 and IIA1). Locally advanced stage: Women with locally advanced stage (FIGO∗ IB2, IIA2 and, IIB). Abbreviations: HR, Hazard Ratio; CI, Confidence Interval, RFS, Recurrence Free Survival; OS, Overall Survival; TMMR, Total MesoMetrial Resection. ∗Staging according to International Federation of Obstetrics and Gynecology, FIGO, 2009 staging manual.

In the total study population, RFS at five years was 82.6% (95% CI 77.2–86.9) and 77.9% (95% CI 74.3–81.1) in the TMMR and ST cohorts, respectively (p = 0.053, Fig. 2). No significant differences in cumulative incidence of local or distant recurrences between the cohorts were observed. In addition, no difference in RFS was observed in the adjusted regression analysis (HR 0.78; 95% CI 0.55–1.10, Fig. 3) or the inverse probability weighting analysis (HR 0.71, 95% 0.51–1.01, Supplementary data). After propensity score matching, borderline significance favoring TMMR was observed (HR 0.72; 95% CI 0.51–1.00, Supplementary data).

In early-stage cervical cancer, RFS at five years was 91.2% (95% CI 85.4–94.9) and 81.8% (95% CI 77.1–85.7) in the TMMR and ST cohorts, respectively (p = 0.002, Fig. 1). The absolute risk difference between TMMR and ST was 9.4% (95% CI 3.2–15.7) (Supplement Table S1). The cumulative incidence of local recurrences was 3.6% and 12.7% for the TMMR and ST cohorts, respectively (sHR0.27; 0.11–0.63, p = 0.003). No significant difference was observed for distal failures. In regression analyses, significant differences in both RFS (HR 0.39; 95% CI 0.22–0.69) and local recurrences (HR 0.26; 95% CI 0.11–0.64) favoring the TMMR cohort, were observed. There was no difference in distant recurrences between the groups (Fig. 3). To assess the potential impact of surgical approach in the Swedish cohort, a sensitivity analysis was performed regarding minimally invasive surgery and open surgery. No significant differences in RFS or OS were observed (Supplement Figure S1).

In locally advanced disease, RFS at five years was 67.4% (95% CI 56.6–76.1) and 72.3% (95% CI 66.4–77.4) in the TMMR and ST cohorts, respectively (p = 0.391, Fig. 2). No differences in cumulative incidence of local or distal recurrences were observed. Further, no differences in RFS, local recurrences, distant recurrences were observed when comparing the treatments in adjusted regression analyses. Landmark analysis for immortal time bias did not change the results significantly (Supplementary data).

### Overall survival

We observed a total of 31 deaths (11%) in the TMMR cohort and 96 deaths (13%) in the ST cohort. At five years, OS in the TMMR and ST cohorts were 88.9% (95% CI 84.0–92.4) and 86.2% (95% CI 83.0–88.9), respectively (p = 0.080, Fig. 2). No significant difference in OS in the adjusted regression analysis (HR 0.75, 95% CI 0.48–1.15, Fig. 3) or the propensity score matching analysis (HR 0.70, 95% CI 0.47–1.05, Supplementary data) was observed. In the inversed probability weighting analysis, a significant difference in OS favoring TMMR was observed (HR 0.65, 95% CI 0.43–0.99, Supplementary data).

OS in early-stage was 93.3% (95% CI 87.4–96.5) in the TMMR cohort and 90.3% (95% CI 86.4–93.2) in the ST cohort (p = 0.034, Fig. 2). In the regression analysis of TMMR compared with ST, a significant difference in OS (HR 0.42; 95% CI 0.21–0.86), favoring TMMR was observed (Fig. 3). In locally advanced cervical cancer, no significant differences were observed in OS between TMMR and ST.

## Discussion

In this large observational cohort study, we present data on the oncologic outcomes of two principally different approaches for primary treatment of curable cervical cancer. In agreement with the theory of cancer field surgery, we observed a close to complete pelvic control after TMMR in early-stage cervical cancer with an absolute risk difference in local recurrences of 9% at five years. In addition, a superior survival was observed in comparison to women treated according to generally accepted recommendations. None of the women treated with TMMR received post-operative adjuvant radiation therapy. In women with locally advanced cervical cancer, similar oncologic outcomes were achieved after TMMR without irradiation as compared to women treated with primary chemoradiation. These outcomes are strengthened by the results from propensity score matching and inverse probability weighting analyses, suggesting superior oncologic outcomes after TMMR for the entire population.

With the random spread model, an arbitrarily chosen margin of healthy tissue must be removed or irradiated to limit the risk of leaving occult cancer. Despite many attempts to describe and develop radical hysterectomy anatomically, local treatment failure is frequent. In the phase III-trial comparing surgery with radiation therapy in early-stage cervical cancer by Landoni et al., more than 25% recurrences were observed, and similar results have been presented during the past decades.^15^, ^16^, ^17^ However, local control after radical hysterectomy appears to have improved over time, which may be the result of improved diagnostics leading to a better selection of women with true early-stage disease.^18^

In this study, we demonstrate that cancer field surgery alone is associated with superior RFS compared to treatment according to current guidelines. In addition, 28% of women treated conventionally received adjuvant radiation therapy with or without concurrent chemotherapy. The perceived benefit of adjuvant radiation therapy is primarily based on a small number of outdated studies and two phase III-trials. In the GOG92 trial, 277 women with early-stage cervical cancer with certain postoperative risk factors, were randomised to either observation or pelvic irradiation after initial radical hysterectomy.^15^ Significant results favouring the radiation therapy arm was reported with 13% locoregional recurrences compared to 19% in the observation arm but with no improvement of overall survival. In the phase III-trial by Peters, 243 women with early-stage cervical cancer (94% stage IB1) with positive pelvic nodes (and/or parametrial involvement) were randomised to either radiation therapy alone or in combination with chemotherapy.^19^ The progression-free survival was 80% in the combined treatment arm *vs* 63% for radiation therapy alone. In the current study, women with early-stage cervical cancer treated with TMMR without adjuvant radiation (regardless of nodal or mesometrial involvement) had a RFS of 90% and a cumulative incidence of locoregional recurrence of less than 4% at five years. The outcomes after conventional treatment were similar to previous studies with a RFS of 82% and 12% locoregional recurrences. This striking difference can only be explained by the fundamentally different view of cancer progression that cancer field surgery represents.

The similar outcomes for locally advanced stages between the two approaches in this study can also be explained by the cancer field model. The in-depth pathoanatomical analysis of the locoregional failures after TMMR with tLND treatment for cervical cancer FIGO (2009) stages IB-IIB has fully complied with the cancer field model demonstrating that local relapses occur only if the cancer field exceeds the surgical treatment field. These situations refer to FIGO IIB tumours of ontogenetic stages >2 as the potential cancer field is not completely removed although the tumours are resected R0. Patients with these disease stages may benefit from primary chemoradiation therapy covering the complete cancer field up to ontogenetic stage oT3b. Unfortunately, current preoperative diagnostics cannot discriminate between oT2 and oT > 2 FIGO IIB cancers with sufficient accuracy. However, according to these arguments, patients with tumours confined to the cervix, including (FIGO 2009) IB2, should be treated with cancer field surgery. Likewise, preserving a functional vagina implies a risk for local recurrences as it originates from the Mullerian system. However, complete removal would have a profound impact on sexual function, and the trade-off appears reasonable considering the low risk of isolated local recurrences.

As postoperative adjuvant radiation therapy was completely withheld in the TMMR group, the anatomical location of metastatic lymph nodes missed at therapeutic lymph node dissection may provide valuable information of potential sites at risk. However, a detailed analyses of treatment failures and subsequent salvage therapy is beyond the scope of the present study.

In the standard treatment group, a majority of women underwent minimally invasive surgery. A recent international multicentre phase III-trial demonstrated worse survival after minimally invasive surgery compared to laparotomy.^18^ Concerns may therefore be raised whether current outcomes may be attributed to surgical approach. In the sensitivity analyses taking surgical approach into account, no difference was observed between open and minimally invasive surgery for neither RFS nor OS, corroborating several large population-based studies in the recent years.^20^, ^21^, ^22^ Ongoing phase III-trials will provide further evidence on the role of minimally invasive surgery for cervical cancer within the next few years.^23^^,^^24^

Our study is limited mainly by its observational design that precludes causal conclusion on the efficacy of the novel treatment strategy (TMMR without adjuvant radiation therapy), moreover, leaving possibilities for selection bias, unknown confounding, and loss to follow-up. These concerns have been addressed to the best of our ability. The registries used to identify the participants minimizes risk of selection bias since the entire population of patients is captured in the Swedish population registries and all patients treated with TMMR in Leipzig were meticulously registered, nevertheless, the registries represent two different healthcare settings. Moreover, the treatment strategy for locally advanced disease precludes surgical-pathological verification of tumour stage in the ST group and upstaging based on suspected lymph node metastases on imaging *vs* histological verification must therefore be interpreted with caution. In addition, our study cannot provide a direct comparison of the treatment-related short- and long-term morbidity of TMMR and ST as the information for the latter is only available in the SQRGC for surgically treated women. However, there was no loss to follow-up, the exposure variable investigated (TMMR) and the outcomes (recurrence, survival) are accurate. Finally, a type II-error cannot be ruled out in the analyses of locally advanced cervical cancer due to the moderate sample size.

The cancer field model is based on more than two decades of preclinical and clinical research. However, the acceptance of TMMR has been surprisingly restrained. A similar development can be traced back to the introduction of total mesorectal excision (TME) for rectal cancer in the early 1980s, which later revolutionized surgical treatment of rectal cancer with a dramatic decline in local recurrence rate.^25^ However, substantial educational efforts were necessary to disseminate the technique and a similar approach is necessary for universal acceptance and implementation of TMMR.^26^

In conclusion, this observational cohort study suggests that TMMR with tLND abandoning postoperative adjuvant radiation therapy may replace the standard treatment approach in early-stage cervical cancer and furthermore be evaluated as an option in locally advanced cervical cancer confined to the Müllerian compartment. Notwithstanding that randomised controlled trials are demanded to precede the implementation of new clinical treatments, it is questionable whether this is justified if the control arm is based on inconsistent or flawed concepts.

## Contributors

HF, AN, SS, MH, and BA conceptualised the study, interpreted data, and wrote the original draft. EA, LW, ND, and BW curated the data. HF, AN, and SS did the formal analysis. HF and SS acquired funding. All authors contributed to the data collection and had permission to access the data. HF, SS, MH, and BA were responsible for project supervision. HF, AN, EA, LW, ND and BW verified the underlying data. All authors reviewed and approved the final manuscript and had final responsibility for the decision to submit for publication.

## Data sharing statement

De-identified data that underlie the results can be shared with other researchers on request by contacting the corresponding author.

## Declaration of interests

HF is a board member of Surgical Science Inc. No other author declared any conflict of interest.
