# Supplementary material for: Oncologic outcomes after Total Mesometrial Resection (TMMR) or treatment according to current international guidelines in FIGO (2009) stages IB1-IIB cervical cancer: an observational cohort study
Source: eClinicalMedicine. 2024 Jun 20;73:102696. doi: 10.1016/j.eclinm.2024.102696 (PMC11245980; doi:10.1016/j.eclinm.2024.102696)
Supplement: Supplementary Figures and Tables [file mmc1.pdf]

Supplementary appendix

Oncologic outcomes after Total Mesometrial Resection (TMMR) or treatment according to current international guidelines in FIGO (2009) stages IB1-IIB cervical cancer: an observational cohort study

Table of contents

Table 1	Crude survival estimates and absolute risk differences between TMMR and ST
Table 2	Multivariate analysis of confounders in Cox regression models
Table 3a	Control of confounding by regression, propensity score or inverse probability weights for any event
Table 3b	Control of confounding by regression, propensity score or inverse probability weights for 1st event local recurrence
Table 3c	Control of confounding by regression, propensity score or inverse probability weights for 1st event distant recurrence
Table 3d	Control of confounding by regression, propensity score or inverse probability weights for 1st event death
Table 3e	Control of confounding by regression, propensity score or inverse probability weights for death
Table 4	Adjusted landmark estimates of the association between TMMR and ST and time to different failures.
Figure 1	Oncologic outcomes by surgical approach in the Swedish cohort

Sub-group	Outcome	TMMR (95% CI)	ST (95% CI)	Diff (%)	Diff lower (%)	Diff upper (%)
All	RFS	82·6 (77·2-86·9)	77·9 (74·3-81·1)	4·7	-1·2	10·6
All	1st LR	8·2 (5·3-12·0)	11·9 (9·5-14·6)	-3·7	-8·0	0·6
All	1st DR	6·8 (4·1-10·4)	7·9 (5·8-10·2)	0·8	-4·9	2·8
All	1st Death	2·3 (0·9-5·1)	2·3 (1·3-4·0)	0·0	-2·4	2·5
All	OS	88·9 (84·0-92·4)	86·2 (83·0-88·9)	2·7	-2·4	7·7
Early	RFS	91·3 (85·4-94·9)	81·8 (77·1-85·7)	9·4	3·2	15·7
Early	1st LR	3·6 (1·5-7·3)	12·7 (9·4-16·5)	-9·0	-13·6	-4·5
Early	1st DR	3·4 (1·3-7·3)	3·9 (2·2-6·5)	-0·5	-4·2	3·1
Early	1st Death	1·7 (0·3-5·5)	1·6 (0·5-3·9)	0·1	-2·7	2·9
Early	OS	93·3 (87·4-96·5)	90·3 (86·4-93·2)	2·9	-2·5	8·4
LACC	RFS	67·4 (56·6-76·1)	72·3 (66·4-77·4)	-4·9	-16·1	6·3
LACC	1st LR	16·4 (9·6-24·8)	11·2 (7·8-15·3)	5·2	-3·4	13·7
LACC	1st DR	12·7 (6·9-20·3)	13·2 (9·3-17·7)	-0·5	-8·4	7·4
LACC	1st Death	3·5 (0·9-9·1)	3·3 (1·5-6·2)	0·2	-4·3	4·8
LACC	OS	81·1 (70·8-88·1)	81·0 (75·6-85·4)	0·1	-9·7	9·8

Table 1 Crude survival estimates and absolute risk differences between TMMR and ST
Early=early-stage cervical cancer (FIGO2009 IB1 and IIA1); LACC=locally advanced cervical cancer (FIGO2009 IB2 and IIB); RFS=recurrence-free survival at 5 years; 1st LR=first event local recurrence; 1st DR=first event distant recurrence; 1st death=first event death; TMMR=total mesometrial resection; ST=standard treatment, CI=confidence interval

	FAIL	1stLR	1stDM	1stDEATH	DEATH
**TMMR**	**0.78**<br>**[0.55,1.10]**<br>**0.152**	**0.69**<br>**[0.41,1.16]**<br>**0.160**	**0.88**<br>**[0.50,1.53]**<br>**0.648**	**1.15**<br>**[0.43,3.05]**<br>**0.778**	**0.75**<br>**[0.48,1.15]**<br>**0.188**
IB2	0.94<br>[0.53,1.67]<br>0.841	0.97<br>[0.46,2.06]<br>0.935	0.87<br>[0.31,2.44]<br>0.792	1.17<br>[0.20,6.85]<br>0.865	1.02<br>[0.49,2.12]<br>0.958
IIA1	2.35<br>[0.83,6.66]<br>0.108	1.45<br>[0.30,7.12]<br>0.646	2.48<br>[0.54,11.39]<br>0.243	2.73<br>[0.26,28.70]<br>0.402	4.12<br>[1.46,11.67]<br>0.008
IIA2	1.84<br>[0.97,3.48]<br>0.060	1.77<br>[0.78,4.01]<br>0.173	1.49<br>[0.44,4.99]<br>0.519	2.47<br>[0.46,13.10]<br>0.289	2.41<br>[1.14,5.08]<br>0.021
IIB	1.04<br>[0.67,1.61]<br>0.864	0.62<br>[0.32,1.18]<br>0.145	1.67<br>[0.78,3.56]<br>0.187	1.64<br>[0.47,5.79]<br>0.441	1.10<br>[0.62,1.93]<br>0.751
Age	1.01<br>[1.00,1.02]<br>0.216	1.00<br>[0.99,1.02]<br>0.712	1.00<br>[0.98,1.02]<br>0.971	1.04<br>[1.01,1.07]<br>0.003	1.01<br>[1.00,1.03]<br>0.037
Year of treatment	0.94<br>[0.89,1.00]<br>0.041	0.94<br>[0.87,1.02]<br>0.144	0.93<br>[0.85,1.03]<br>0.153	0.87<br>[0.72,1.05]<br>0.147	0.93<br>[0.86,1.00]<br>0.066
Adeno,AdenoSquam	1.55<br>[1.13,2.13]<br>0.007	2.41<br>[1.56,3.72]<br>0.000	0.94<br>[0.53,1.67]<br>0.830	0.27<br>[0.06,1.22]<br>0.088	1.59<br>[1.05,2.39]<br>0.027
Lymph node metastases	1.91<br>[1.39,2.64]<br>0.000	2.19<br>[1.37,3.50]<br>0.001	1.70<br>[1.02,2.85]<br>0.041	1.20<br>[0.52,2.78]<br>0.662	2.03<br>[1.39,2.98]<br>0.000
Tumour size	1.68<br>[1.29,2.18]<br>0.000	1.51<br>[1.05,2.17]<br>0.025	2.14<br>[1.35,3.38]<br>0.001	1.08<br>[0.53,2.20]<br>0.832	1.64<br>[1.17,2.30]<br>0.004
N	991	991	991	991	991
E	191	100	67	24	126
CE	0	91	124	167	0

Table 2. All patients 2011-2020: Adjusted estimates of the association between TMMR and Standard treatment and time to different failures.

Competing risk regression (Fine and Gray) or proportional hazards regression (Cox): sHR/HR, 95% CI and Wald p-values.

Treatment reference category= Standard.

E= Total number of events. CE= Total number of competing events.

	UNADJ	**REGR**	PS	IPW
TMMR	0.72<br>[0.52,1.00]<br>0.053	0.78<br>[0.55,1.10]<br>0.152	0.72<br>[0.51,1.00]<br>0.049	0.71<br>[0.51,1.01]<br>0.054

Table 3a. All patients Event FAIL (any event): Control of confounding by regression, propensity score or inverse probability weights.

Proportional hazards regression (Cox): HR, 95%CI.

Treatment reference category = Standard.

Confounders: age, year of treatment, stage, histology, positive lymph nodes and tumor size.

UNADJ= Only treatment included in the regression model (crude effect).

REGR= Confounders included in the regression model.

PS= Confounders included in the regression model as quintiles of the propensity score.

IPW= Confounders included using inverse probability weights and included in the regression model as weights.

	UNADJ	**REGR**	PS	IPW
TMMR	0.62<br>[0.39,1.01]<br>0.055	0.69<br>[0.41,1.16]<br>0.160	0.67<br>[0.41,1.09]<br>0.105	0.69<br>[0.42,1.13]<br>0.141

Table 3b. All patients Event 1st local recurrence: Control of confounding by regression, propensity score or inverse probability weights.
Proportional hazards regression (Cox): HR, 95%CI.
Treatment reference category = Standard.
Confounders: age, year of treatment, stage, histology, positive lymph nodes and tumor size.
UNADJ= Only treatment included in the regression model (crude effect).
REGR= Confounders included in the regression model.
PS= Confounders included in the regression model as quintiles of the propensity score.
IPW= Confounders included using inverse probability weights and included in the regression model as weights.

	UNADJ	**REGR**	PS	IPW
TMMR	0.85<br>[0.50,1.46]<br>0.561	0.88<br>[0.50,1.53]<br>0.648	0.78<br>[0.45,1.34]<br>0.364	0.77<br>[0.44,1.33]<br>0.344

Table 3c. All patients Event 1st distant recurrence: Control of confounding by regression, propensity score or inverse probability weights.
Proportional hazards regression (Cox): HR, 95%CI.
Treatment reference category = Standard.
Confounders: age, year of treatment, stage, histology, positive lymph nodes and tumor size.
UNADJ= Only treatment included in the regression model (crude effect).
REGR= Confounders included in the regression model.
PS= Confounders included in the regression model as quintiles of the propensity score.
IPW= Confounders included using inverse probability weights and included in the regression model as weights.

	UNADJ	**REGR**	PS	IPW
TMMR	1.03<br>[0.44,2.41]<br>0.951	1.15<br>[0.43,3.05]<br>0.778	0.98<br>[0.42,2.29]<br>0.964	0.90<br>[0.38,2.15]<br>0.811

Table 3d. All patients Event 1st DEATH: Control of confounding by regression, propensity score or inverse probability weights.
Proportional hazards regression (Cox): HR, 95%CI.
Treatment reference category = Standard.
Confounders: age, year of treatment, stage, histology, positive lymph nodes and tumor size.
UNADJ= Only treatment included in the regression model (crude effect).
REGR= Confounders included in the regression model.
PS= Confounders included in the regression model as quintiles of the propensity score.
IPW= Confounders included using inverse probability weights and included in the regression model as weights.

	UNADJ	**REGR**	PS	IPW
TMMR	0.70<br>[0.47,1.04]<br>0.080	0.75<br>[0.48,1.15]<br>0.188	0.70<br>[0.47,1.05]<br>0.084	0.65<br>[0.43,0.99]<br>0.042

Table 3e. All patients Event DEATH: Control of confounding by regression, propensity score or inverse probability weights.

Proportional hazards regression (Cox): HR, 95%CI.
Treatment reference category = Standard.
Confounders: age, year of treatment, stage, histology, positive lymph nodes and tumor size.
UNADJ= Only treatment included in the regression model (crude effect).
REGR= Confounders included in the regression model.
PS= Confounders included in the regression model as quintiles of the propensity score.
IPW= Confounders included using inverse probability weights and included in the regression model as weights.

	FAIL	1stLR	1stDM	1stDEATH	DEATH
TMMR	1.34<br>[0.87,2.08]<br>0.188	1.72<br>[0.86,3.46]<br>0.128	0.94<br>[0.47,1.88]<br>0.851	1.70<br>[0.55,5.20]<br>0.353	1.09<br>[0.62,1.91]<br>0.769
N	369	369	369	369	369
E	109	48	46	15	75
CE	0	61	63	94	0

Table 4. Locally advanced stage patients 2011-2020: Adjusted landmark (+180 days) estimates of the association between country and time to different failures.
Competing risk regression (Fine and Gray) or proportional hazards regression (Cox)): sHR/HR, 95%CI and Wald p-values.
Reference category = Standard treatment
E= Total number of events. CE= Total number of competing events.
All patients alive and recurrence-free at landmark (180 days).
Adjusted for age, year of treatment, stage, histology, positive lymph nodes and tumor size.

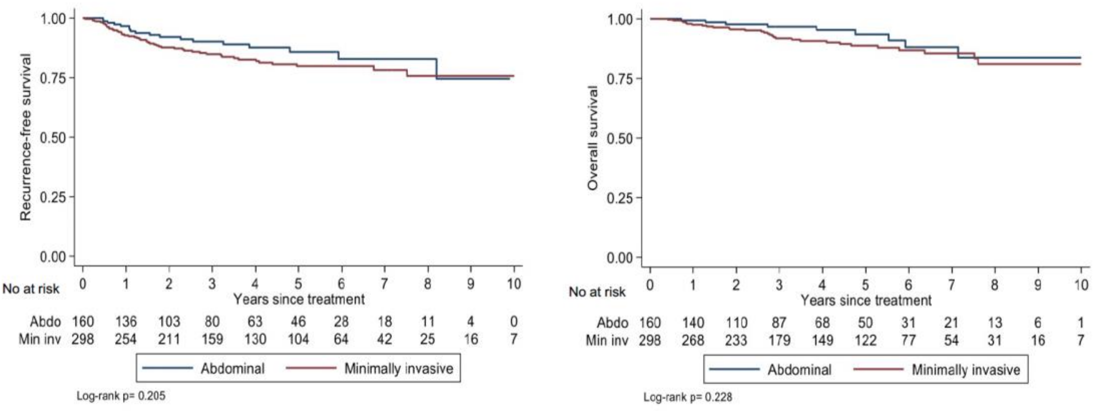

Figure 1 RFS and OS in women with early-stage (FIGO* IB1, IIA1) cervical cancer in the Swedish cohort, by minimally invasive surgery or laparotomy
Blue line: Abdominal surgery. Red line: Minimally invasive surgery.
FIGO=International Federation of Gynecology and Obstetrics;
*Staging according to International Federation of Obstetrics and Gynecology, FIGO, 2009 staging manual
